# Neuroimmune regulation of visceral pain: roles of microglia, purinergic signaling, and neuroinflammation

**DOI:** 10.3389/fimmu.2026.1934162

**Published:** 2026-08-27

**Authors:** Yu Wang, Peizhe Li, Yanan Dong, Xin Zhang, Kun Shang

**Affiliations:** Department of Acupuncture and Massage, Changchun University of Chinese Medicine, Jilin, China

**Keywords:** COMSOL multiphysics, irritable bowel syndrome, neuroimmune crosstalk, spinal microglia, visceral hypersensitivity

## Abstract

Visceral hypersensitivity is a hallmark pathophysiological feature of irritable bowel syndrome, characterized by enhanced perception of visceral stimuli and a reduced pain threshold. Although previous studies have mainly attributed visceral hypersensitivity to peripheral inflammation, visceral afferent sensitization, and psychological stress, these mechanisms do not fully explain the persistent pain and discomfort observed in some patients without overt organic lesions or pronounced inflammation. Accumulating evidence indicates that the spinal cord is not merely a relay site for visceral sensory afferent input, but also a critical central locus for pain modulation and amplification. As resident immune cells of the central nervous system, spinal microglia can be activated by persistent visceral stimulation, inflammation, and stress, thereby contributing to the development and maintenance of visceral hypersensitivity through neuroimmune crosstalk. This review summarizes the role of spinal microglia in the development and progression of visceral hypersensitivity and the associated molecular mechanisms. For the first time in this research field, a two-dimensional reaction-diffusion numerical model was established using COMSOL Multiphysics to simulate the spatiotemporal diffusion and degradation patterns of key signaling molecules, including CSF1, ATP, and BDNF, within the microenvironment of the spinal dorsal horn. The model further incorporates coupled analyses of dynamic changes in microglial activation states and neuronal membrane responses, providing a theoretical basis for quantitatively elucidating microglia-mediated pain sensitization. Evidence indicates that signaling molecules such as ATP, CX3CL1, and CSF1 can drive the activation of microglia in the spinal dorsal horn and induce the release of mediators including TNF-α, IL-1β, and BDNF. These changes enhance excitatory synaptic transmission, weaken inhibitory neurotransmission, and promote spinal and central sensitization. Among these mechanisms, the BDNF-TrkB-KCC2/NMDAR signaling axis represents a key molecular pathway underlying microglia-mediated pain amplification. Furthermore, peripheral factors, including intestinal inflammation, psychological stress, and gut microbiota dysbiosis, may further regulate spinal microglial reactivity through the gut-brain axis, thereby exacerbating visceral hypersensitivity. This review not only summarizes recent advances in understanding the involvement of spinal microglia in visceral hypersensitivity, but also provides a theoretical basis for mechanistic studies and targeted interventions in other neuroinflammation-related pathological processes and chronic pain disorders.

## 1 Introduction

Visceral hypersensitivity (VH) is one of the hallmark pathophysiological features of disorders of gut-brain interaction (DGBI), particularly irritable bowel syndrome (IBS) (1–3). VH is primarily characterized by a reduced threshold for visceral sensation and an abnormally heightened responsiveness to gastrointestinal stimuli. Clinically, it may manifest as allodynia, in which pain is evoked by normally innoxious stimuli, and hyperalgesia, in which pain responses to noxious stimuli are markedly enhanced (4–6). This abnormal transmission and modulation of visceral sensation renders patients more sensitive to intestinal distension, chemical stimulation, and inflammation-related signals, and constitutes an important pathophysiological basis for the persistence of abdominal pain and abdominal discomfort in patients with IBS (7, 8). Epidemiological data indicate that, based on the Rome III criteria, the global prevalence of IBS is approximately 10.1%, whereas under the more stringent Rome IV diagnostic criteria, the global prevalence is approximately 3.8% (9–11). Owing to its high prevalence, chronic disease course, and substantial negative impact on quality of life, IBS has become a major public health concern worldwide. Although previous studies have extensively investigated the mechanisms underlying VH from the perspectives of peripheral inflammation, sensitization of visceral afferent nerves, and psychological stress, these factors remain insufficient to fully explain the persistent pain and discomfort observed in some patients in the absence of overt organic lesions or severe inflammation. Specifically, they fail to account for the persistence of VH in IBS patients without obvious inflammation, suggesting that central regulatory mechanisms may play a key role in the development and chronification of VH. Therefore, current evidence is still insufficient to comprehensively explain the onset and persistence of VH, and novel underlying mechanisms remain to be further elucidated. In particular, the biological basis for symptom persistence, frequent relapse, and the tendency toward chronicity in a subset of patients requires further in-depth investigation.

In recent years, increasing evidence has shown that the neuroimmune system plays an important role in visceral hypersensitivity, particularly the interactions between the central nervous system (CNS) and immune cells, which have received growing attention for their involvement in the development and maintenance of chronic pain (12, 13). An in-depth investigation of neuroimmune crosstalk in visceral hypersensitivity may help clarify how peripheral gastrointestinal disturbances induce central sensitization and chronic visceral pain, reveal the underlying pathogenesis of this condition, and identify potential novel targets for clinical treatment. In this context, the inflammatory reflex provides an important conceptual framework for understanding bidirectional neuroimmune regulation. As originally described in Kevin Tracey’s seminal work, vagus nerve-mediated neural circuits can regulate peripheral inflammatory responses and thereby influence gut-brain communication and visceral sensory processing (14, 15). Although this reflex highlights the systemic regulation of inflammation through autonomic pathways, visceral nociceptive information still requires integration and modulation within the CNS before contributing to persistent hypersensitivity and pain. Therefore, in addition to vagal neuroimmune modulation, spinal mechanisms are also essential for understanding how peripheral inflammatory and sensory signals are translated into central sensitization. The spinal cord is a key integrative hub for visceral sensory information after it enters the CNS, as well as an important site for pain signal transmission and modulation (16, 17). Abnormal peripheral stimuli require preliminary integration at the spinal level, and because the spinal cord is also a critical node in the formation of central sensitization, it may play a key role in the development and maintenance of VH. Within the local spinal cord microenvironment, microglia, as resident immune cells of the CNS, constitute an important component of neuroimmune crosstalk and play critical roles in maintaining central homeostasis, monitoring changes in neuronal activity, and regulating local inflammatory responses (18, 19). Under physiological conditions, microglia predominantly remain in a dynamic surveillant state, continuously sensing changes in surrounding neurons, synapses, and other glial cells through their processes, and participating in processes such as synaptic pruning, clearance of metabolic waste, and maintenance of neural network stability (20, 21). Under pathological conditions, such as persistent visceral stimulation, peripheral inflammation, or psychological stress, aberrant sensory input may disrupt homeostasis in the local spinal cord microenvironment and induce a transition of microglia from a surveillant state to an activated state, characterized by changes in morphology, phenotype, and function (22, 23). Because the spinal dorsal horn is a major site for the integration of peripheral visceral sensory information after it enters the CNS, as well as a key region for pain signal transmission and modulation, activation of microglia in this region may exert an important influence on the amplification and persistence of abnormal visceral sensory signals. In recent years, accumulating evidence has suggested that activation of microglia in the spinal dorsal horn is closely associated with the development and maintenance of VH. Activated microglia may contribute to spinal sensitization and central sensitization by regulating local inflammatory responses, modulating neuronal excitability, and altering the efficiency of synaptic transmission (24–26). Therefore, microglia are not merely immune effector cells; rather, they may represent a critical link connecting aberrant peripheral stimuli, local neuroimmune responses in the spinal cord, and central amplification of pain signaling, and thus constitute an important research target for elucidating the pathogenesis of VH.

In this review, we systematically summarize the role of spinal microglia in the development and maintenance of VH. Furthermore, a COMSOL Multiphysics-based simulation approach is incorporated to analyze the diffusion behavior and potential spatial influence of representative inflammatory mediators within the local microenvironment of the spinal dorsal horn. Through the construction of a local tissue microenvironment model, the effects of diffusion parameters, degradation conditions, and release sites on inflammatory mediator concentration profiles are examined. This spatial-scale perspective may provide a useful reference for understanding the regulatory effects of microglia-derived factors on neighboring neuronal activity and may offer new insights for future interventional studies targeting neuroinflammation associated with visceral pain.

## 2 Neuron-microglia communication in spinal central sensitization

Under conditions of chronic pain, bidirectional communication between neurons and microglia has emerged as a central mechanism mediating the initiation and progression of central sensitization. In the spinal dorsal horn, neurons and microglia form a dynamic signaling network: Neuronal activity can rapidly trigger microglial responses, while activated microglia, in turn, modulate neuronal excitability and synaptic transmission. This interaction plays a critical role in amplifying nociceptive signaling and maintaining persistent hyperalgesia (27, 28). Under physiological conditions, microglia remain in a surveillant state, with their highly ramified processes continuously scanning the surrounding neural microenvironment. In response to neuronal injury, inflammation, or persistent nociceptive stimulation, neurons release a variety of signaling molecules that serve as initiating signals for microglial activation. These neuron-derived signals can initiate intracellular signaling cascades in microglia, promoting morphological remodeling, proliferation, and the production of pro-inflammatory mediators (29).

The most extensively studied signaling pathways mediating neuron-microglia communication include purinergic signaling, the fractalkine/CX3CL1-CX3CR1 signaling pathway, and the colony-stimulating factor 1 (CSF1)-colony-stimulating factor 1 receptor (CSF1R) signaling pathway. During neuronal excitation or cellular stress, adenosine triphosphate (ATP) is released into the extracellular space, where it acts as an important damage-associated molecular pattern (DAMP) signal to activate microglia (30). Microglia express multiple purinergic receptors on their surface, including P2X4, P2X7, and P2Y12 receptors, which recognize extracellular ATP and initiate microglial activation (31–33). These receptors play distinct yet interrelated roles at different stages of spinal central sensitization. The P2Y12 receptor is a G protein-coupled receptor that primarily detects ADP signals generated from ATP metabolism and plays an important role in the early microglial response to aberrant neuronal activity. Activation of the P2Y12 receptor promotes the directed extension of microglial processes toward active neurons or sites of injury, enhances chemotactic migration and surveillance of changes in the synaptic microenvironment, and thereby facilitates dynamic contact and functional coupling between neurons and microglia (34–36). The P2X4 receptor is an ATP-gated ion channel, and its activation can induce the release of BDNF from microglia. BDNF subsequently acts on TrkB receptors expressed by dorsal horn neurons, altering neuronal chloride homeostasis and weakening inhibitory synaptic transmission. This ultimately increases the excitability of dorsal horn neurons and promotes the amplification of nociceptive signaling (37, 38). This mechanism contributes to the disinhibition of spinal nociceptive circuits and drives the development of central sensitization.

CX3CL1 is predominantly expressed by neurons, whereas its receptor, CX3CR1, is selectively expressed on microglia within the CNS (39, 40). Under conditions of neuronal stress or inflammation, membrane-bound CX3CL1 can be cleaved and released as a soluble chemokine, thereby activating CX3CR1 signaling in microglia (41). Activation of this pathway promotes microglial proliferation and the production of pro-inflammatory cytokines, including tumor necrosis factor-α (TNF-α) and interleukin-1β (IL-1β) (42). These cytokines can directly enhance synaptic transmission in dorsal horn neurons and amplify nociceptive information processing by potentiating glutamatergic signaling and facilitating activation of N-methyl-D-aspartate receptors (NMDARs) (43).

CSF1 signaling is also involved in neuron-microglia communication within pain pathways. Following peripheral nerve injury, primary sensory neurons can release CSF1, which binds to colony-stimulating factor 1 receptor expressed on the surface of microglia. This signaling pathway promotes microglial proliferation and drives their polarization toward a pro-inflammatory phenotype. The resulting increase in microglial density in the spinal dorsal horn further amplifies neuroimmune signaling and contributes to the maintenance of persistent hyperalgesia (44).

Activated microglia can also profoundly influence neuronal function through the release of inflammatory mediators and neuroactive substances. Cytokines such as TNF-α, interleukin-1β (IL-1β), and interleukin-6 (IL-6) can enhance excitatory synaptic transmission by increasing presynaptic glutamate release and facilitating postsynaptic receptor activation (45). In parallel, these cytokines can modulate intracellular signaling pathways involved in synaptic plasticity, including the mitogen-activated protein kinase (MAPK) and nuclear factor-κB (NF-κB) pathways, thereby further regulating pain-related gene expression and synaptic remodeling (46, 47). In addition, microglia-derived BDNF represents another key mediator linking neuroinflammation to synaptic plasticity (48). BDNF released from activated microglia can alter the expression and function of potassium-chloride cotransporter 2 (KCC2, K^+^-Cl^-^ cotransporter 2) in dorsal horn neurons through activation of TrkB signaling. Downregulation of KCC2 disrupts chloride homeostasis and weakens inhibitory neurotransmission mediated by γ-aminobutyric acid (GABA) and glycine (37, 49, 50). Ultimately, the reduced efficacy of inhibitory synaptic input leads to increased neuronal excitability and enhanced nociceptive signal transmission.

**Figure 1:**
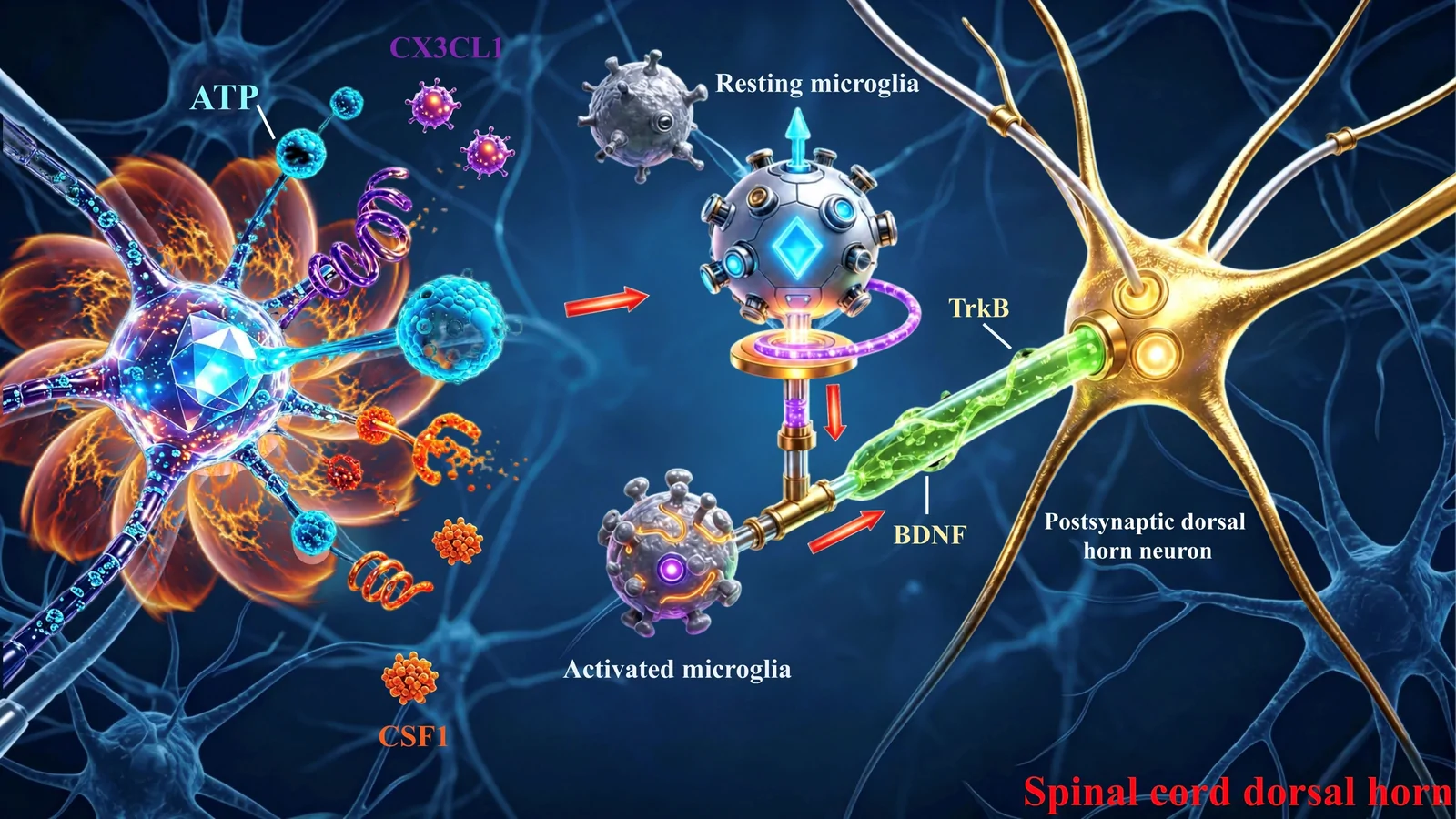
Schematic illustration of neuron-microglia signaling pathways involved in spinal central sensitization. Neuron-derived ATP, CX3CL1, and CSF1 act as upstream signals that activate spinal microglia. Activated microglia subsequently release BDNF, which acts on TrkB receptors in postsynaptic dorsal horn neurons, thereby enhancing neuronal excitability and contributing to central sensitization.

In summary, neuron-microglia signaling forms a feed-forward loop that contributes to the initiation and maintenance of central sensitization in spinal nociceptive pathways. Persistent nociceptive input from peripheral tissues enhances neuronal activity and promotes the release of microglia-activating signals, including ATP, CX3CL1, and CSF1. These signals activate spinal microglia through their corresponding receptors and induce microglial proliferation, phenotypic transformation, and the production of inflammatory mediators. Activated microglia, in turn, release cytokines and neurotrophic factors, such as TNF-α, IL-1β, IL-6, and BDNF, which enhance excitatory synaptic transmission, impair inhibitory control, and promote maladaptive synaptic plasticity in the spinal dorsal horn. The integrated signaling network underlying this neuron-microglia feed-forward loop is summarized in **Figure 1**. Although current understanding of neuron-microglia communication has largely been derived from studies of neuropathic pain, emerging evidence indicates that similar mechanisms are also involved in the pathogenesis of visceral pain disorders. In conditions such as VH, peripheral gastrointestinal inflammation and dysregulated brain-gut signaling can activate spinal nociceptive pathways and initiate microglia-mediated neuroinflammatory responses (51, 52). Therefore, elucidating the molecular mechanisms of neuron-microglia communication in visceral pain pathways may provide an important theoretical basis for understanding the pathogenesis of functional gastrointestinal disorders and help identify novel therapeutic targets for the treatment of chronic visceral pain. The following section therefore focuses on how this general neuron-microglia signaling framework is implemented in visceral pain, with emphasis on visceral afferent-driven microglial activation, pathway-specific mediators, and their contribution to VH.

## 3 Spinal microglia-mediated neuroinflammation in visceral pain

Increasing evidence indicates that neuroinflammation driven by spinal microglial activation is one of the key mechanisms underlying the pathogenesis of chronic pain and may contribute to central sensitization and the development of VH in visceral pain syndromes such as IBS and inflammatory bowel disease (IBD) (26, 53, 54). As resident immune cells of the CNS, microglia not only participate in immune surveillance and maintenance of tissue homeostasis under physiological conditions, but also play a profound role in pathological pain processing by regulating neuronal excitability, synaptic plasticity, and the local inflammatory microenvironment. In particular, in the spinal dorsal horn, persistent visceral nociceptive input can induce microglial activation, thereby triggering local neuroinflammation, enhancing nociceptive synaptic transmission, and weakening inhibitory control (55–57). Therefore, spinal microglial activation is considered one of the core central mechanisms involved in the development and maintenance of VH. This section reviews the major features of spinal microglial activation in the context of visceral pain, the key signaling mediators involved, and its effects on spinal nociceptive circuits.

### 3.1 Spinal microglial activation in neuroinflammation

**Figure 2:**
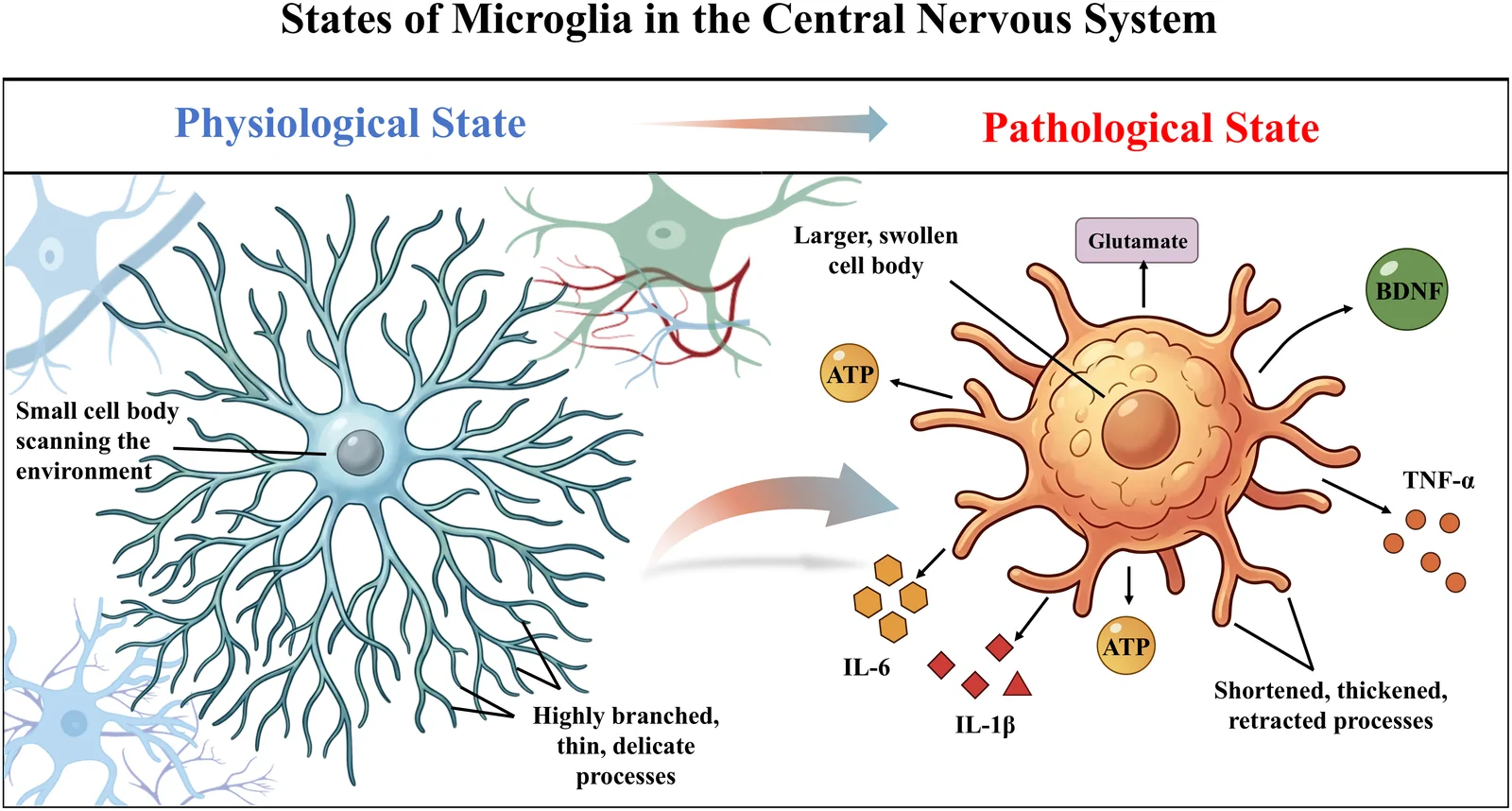
Transition of microglia from a homeostatic surveillance state to an activated neuroinflammatory state in the CNS. Upon pathological stimulation, microglia undergo morphological remodeling, characterized by soma enlargement and process retraction, and release ATP, glutamate, BDNF, TNF-alpha, IL-6, and IL-1beta, which contribute to neuroinflammation and neuronal sensitization.

Under physiological conditions, microglia remain in a homeostatic surveillance state, characterized by small cell bodies and highly ramified processes, and continuously scan the surrounding neural microenvironment to detect potential injury- or inflammation-related signals (58). This surveillance function is essential for maintaining CNS homeostasis, including the clearance of apoptotic cells, mediation of synaptic pruning, and preservation of neuronal connectivity and functional integrity (59). However, when the organism is exposed to harmful stimuli such as peripheral inflammation, tissue injury, or chronic stress, microglia can rapidly transition from a homeostatic state to an activated state, accompanied by marked morphological and functional changes. As shown in **Figure 2**, microglia undergo a transition from a highly ramified surveillance phenotype under physiological conditions to an activated phenotype characterized by enlarged cell bodies, shortened processes, and increased release of pro-inflammatory and neuroactive mediators under pathological conditions. First, microglia undergo morphological remodeling, shifting from a highly ramified surveillance phenotype to an activated phenotype characterized by enlarged cell bodies and shortened, thickened processes, which facilitates their migration toward damaged or inflammation-associated regions. Second, activated microglia proliferate within the affected areas, leading to an increase in local microglial cell numbers and thereby amplifying the neuroinflammatory response (27, 60). Upon activation, microglia release a variety of pro-inflammatory mediators, including cytokines such as TNF-α, IL-1β, and IL-6, chemokines, growth factors, and neuroactive molecules such as ATP (61). These mediators not only regulate neuronal activity locally, but also promote the establishment and maintenance of central sensitization by enhancing synaptic plasticity and neuronal hyperexcitability. In visceral pain and VH, microglial activation occurs predominantly in the spinal dorsal horn, a critical site for the integration of visceral sensory information, where gastrointestinal sensory afferents converge onto spinal nociceptive circuits. Peripheral nociceptive stimuli originating from the gut, such as mucosal inflammation, gut microbiota dysbiosis, and local ischemia, can activate primary afferent neurons and transmit abnormally enhanced nociceptive input to the spinal dorsal horn. These signals serve as triggers for microglial activation, thereby forming a bidirectional communication loop between peripheral injury and central neuroinflammation (26, 55, 62).

### 3.2 Key mediators and signaling pathways of spinal microglial activation in visceral pain

In visceral pain, microglial activation in the spinal dorsal horn can be organized into three hierarchical levels: upstream neuron- or glia-derived activating signals, microglial receptor and intracellular signaling pathways, and downstream mediators that reshape neuronal excitability. ATP/ADP, CX3CL1, and CSF1 mainly function as upstream activating signals. P2 receptors, CX3CR1, and CSF1R serve as microglial sensing receptors, with p38 MAPK, ERK, NF-κB, and inflammasome-related pathways acting as intracellular transducers. Cytokines and neurotrophic factors, including TNF-α, IL-1β, IL-6, and BDNF, then act on dorsal horn neurons to enhance excitatory transmission, impair inhibitory control, and promote central sensitization.

#### 3.2.1 Purinergic signaling: P2 receptor-mediated microglial activation

In visceral pain states, ATP/ADP-dependent purinergic signaling represents a major route through which persistent visceral afferent activity is translated into spinal microglial responses (63). Persistent nociceptive input from visceral organs can enhance ATP release and ADP generation in the spinal dorsal horn. ADP-sensitive P2Y12 receptors are particularly relevant to the early microglial response, promoting process extension and recruitment of microglia toward hyperactive neuronal elements. Recent evidence further indicates that gut-innervating TRPV1-positive sensory neurons can drive chronic visceral pain through spinal microglial P2Y12 signaling, supporting P2Y12 as a molecular link between peripheral visceral input and central neuroimmune activation (25, 36). Compared with P2Y12-mediated microglial recruitment, P2X4 signaling is more closely associated with the effector phase of central sensitization. Activation of P2X4 can induce Ca^2+^-dependent p38 MAPK phosphorylation in microglia, thereby promoting the production of BDNF and pro-inflammatory mediators such as TNF-α, IL-1β, and IL-6 (27, 31, 64–68). In visceral pain, these mediators may enhance NMDAR-mediated excitatory transmission and weaken inhibitory control through the BDNF-TrkB-KCC2 axis, ultimately increasing dorsal horn neuronal responsiveness to visceral afferent input (45, 69–71). In parallel, these cytokines can further activate microglia and astrocytes within the spinal cord, amplifying positive feedback loops between neurons and glial cells and establishing a persistent neuroinflammatory microenvironment (54, 72–74). This process is critical for the transition of visceral pain from a transient stress response to a chronic and persistent state (75, 76).

BDNF is one of the key effector molecules released by activated microglia and serves as an important molecular bridge linking neuroinflammation to aberrant synaptic plasticity (37, 48). After binding to TrkB receptors on neurons in the spinal dorsal horn, BDNF activates downstream signaling pathways, enhances neuronal excitability, and facilitates nociceptive transmission (77). In the spinal cord, BDNF-TrkB signaling can strengthen excitatory synaptic transmission by promoting NMDAR phosphorylation and enhancing NMDAR function, which represents one of the major mechanisms by which BDNF induces central sensitization (78–80). BDNF can also disrupt intracellular Cl^-^ homeostasis by downregulating the expression and function of KCC2 in spinal dorsal horn neurons, thereby weakening GABAergic and glycinergic inhibitory transmission and inducing disinhibition of spinal dorsal horn neurons (37, 50, 81, 82). When inhibitory control is reduced, the spinal gating of visceral sensory input is impaired, allowing low-intensity or even non-nociceptive visceral stimuli to be abnormally amplified and encoded as pain signals, thereby promoting the development and maintenance of VH (78, 83, 84). Thus, BDNF directly enhances NMDAR function on the one hand and indirectly facilitates NMDAR activation by weakening inhibitory control on the other, forming a dual amplification mechanism that promotes spinal sensitization.

**Figure 3:**
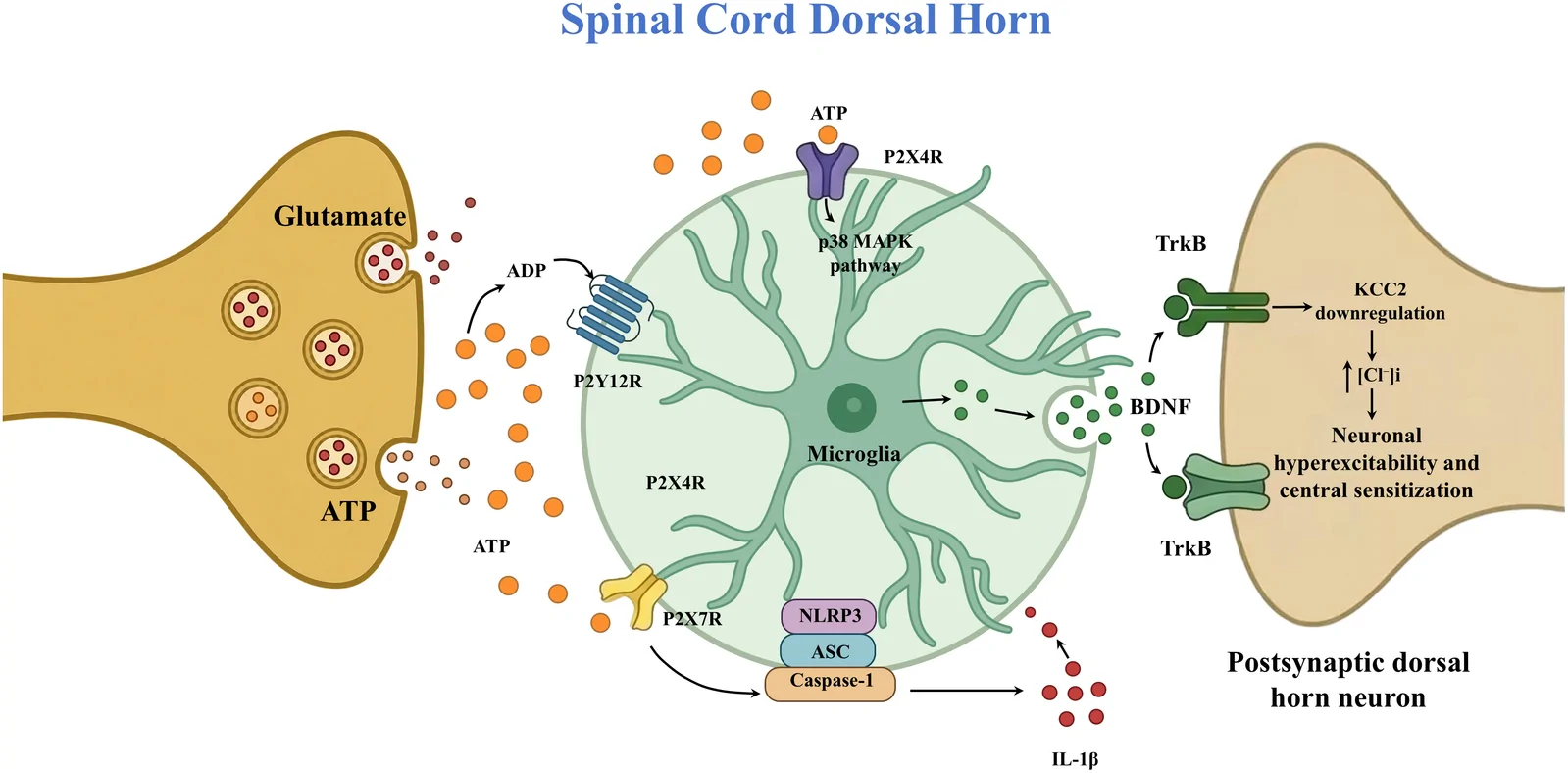
Schematic illustration of ATP/ADP-mediated purinergic signaling pathways linking spinal microglial activation to central sensitization in visceral pain. ATP and ADP released in the spinal dorsal horn activate microglial P2X4R, P2X7R, and P2Y12R; P2X4R-mediated p38 MAPK activation promotes BDNF release and TrkB-dependent KCC2 downregulation in postsynaptic neurons, whereas P2X7R activation induces NLRP3 inflammasome-mediated IL-1beta release, collectively contributing to neuronal hyperexcitability and central sensitization.

P2X7 receptors are also closely associated with inflammasome-related signaling activation and can further amplify neuroinflammatory responses (85, 86). Activation of P2X7 receptors promotes the release of IL-1β, which is considered one of the key pro-inflammatory cytokines involved in spinal central sensitization (22, 73, 87). Meanwhile, P2X7 receptor activation can induce microglia to release various pronociceptive mediators, such as reactive oxygen species and nitric oxide, which further enhance the excitability of dorsal horn neurons through oxidative stress, synaptic modulation, and amplification of inflammatory signaling (88). In addition, P2X7 receptors can activate inflammation-associated signaling pathways, including p38 MAPK, ERK, and NF-κB, thereby promoting the transition of microglia toward a pro-inflammatory effector phenotype and maintaining their chronic activation state. This provides a molecular basis for the persistence of chronic pain and, in particular, VH (46, 89–92). In summary, ATP/ADP-mediated purinergic signaling through P2Y12, P2X4, and P2X7 receptors contributes to the activation and functional sensitization of spinal microglia, thereby promoting microglia-neuron communication, enhancing the excitability of spinal dorsal horn neurons, and facilitating the development and maintenance of central sensitization in visceral pain. As illustrated in **Figure 3**, extracellular ATP/ADP functions as an upstream danger signal, whereas microglial inflammatory mediators and altered neuronal chloride homeostasis represent key downstream mechanisms linking purinergic signaling to visceral pain hypersensitivity.

#### 3.2.2 Neuron and glia-derived signals: CX3CL1-CX3CR1 and CSF1-CSF1R pathways

The fractalkine/CX3CL1-CX3CR1 signaling pathway is considered an important and specific communication axis between neurons and microglia in the CNS (93). CX3CL1 is primarily expressed by neurons and exists in both membrane-bound and soluble forms, whereas its specific receptor CX3CR1 is predominantly expressed by central microglia (94, 95). Therefore, this signaling axis is regarded as an important molecular bridge through which neurons transmit information related to injury, inflammation, and abnormal excitation to microglia. Previous studies have shown that the CX3CL1/CX3CR1 signaling axis can mediate aberrant neuron–microglia communication under conditions of nerve injury and inflammation, and promote neuroinflammation and central sensitization through downstream pathways such as p38 MAPK (40, 42). In models of VH, studies have suggested that spinal cathepsin S may promote VH via the FKN/CX3CR1/p38 MAPK signaling pathway, indicating that this axis may participate in spinal sensitization associated with visceral pain (26). The CX3CL1/CX3CR1 axis may be one of the key nodes in maintaining the positive feedback loop between neurons and microglia. Persistent visceral nociceptive input can promote the cleavage and release of membrane-bound CX3CL1 from spinal dorsal horn neurons, accompanied by increased ATP and other damage-associated signals, thereby activating neighboring microglia. At the same time, enhanced glutamatergic transmission may also jointly promote a hyperexcitable state in dorsal horn neurons. Activated microglia further release sensitizing mediators such as IL-1β, TNF-α, and BDNF, which in turn enhance synaptic transmission and excitability of dorsal horn neurons, amplifying neuron-glia interactions and promoting the maintenance of central sensitization. Previous studies have shown that intervention in CX3CR1/p38 MAPK signaling can alleviate microglial activation, neuroinflammation, and pain hypersensitivity in neuropathic pain, inflammatory pain, and some VH models, suggesting that this pathway may be involved in the chronic progression of visceral pain (40, 54, 96).

**Figure 4:**
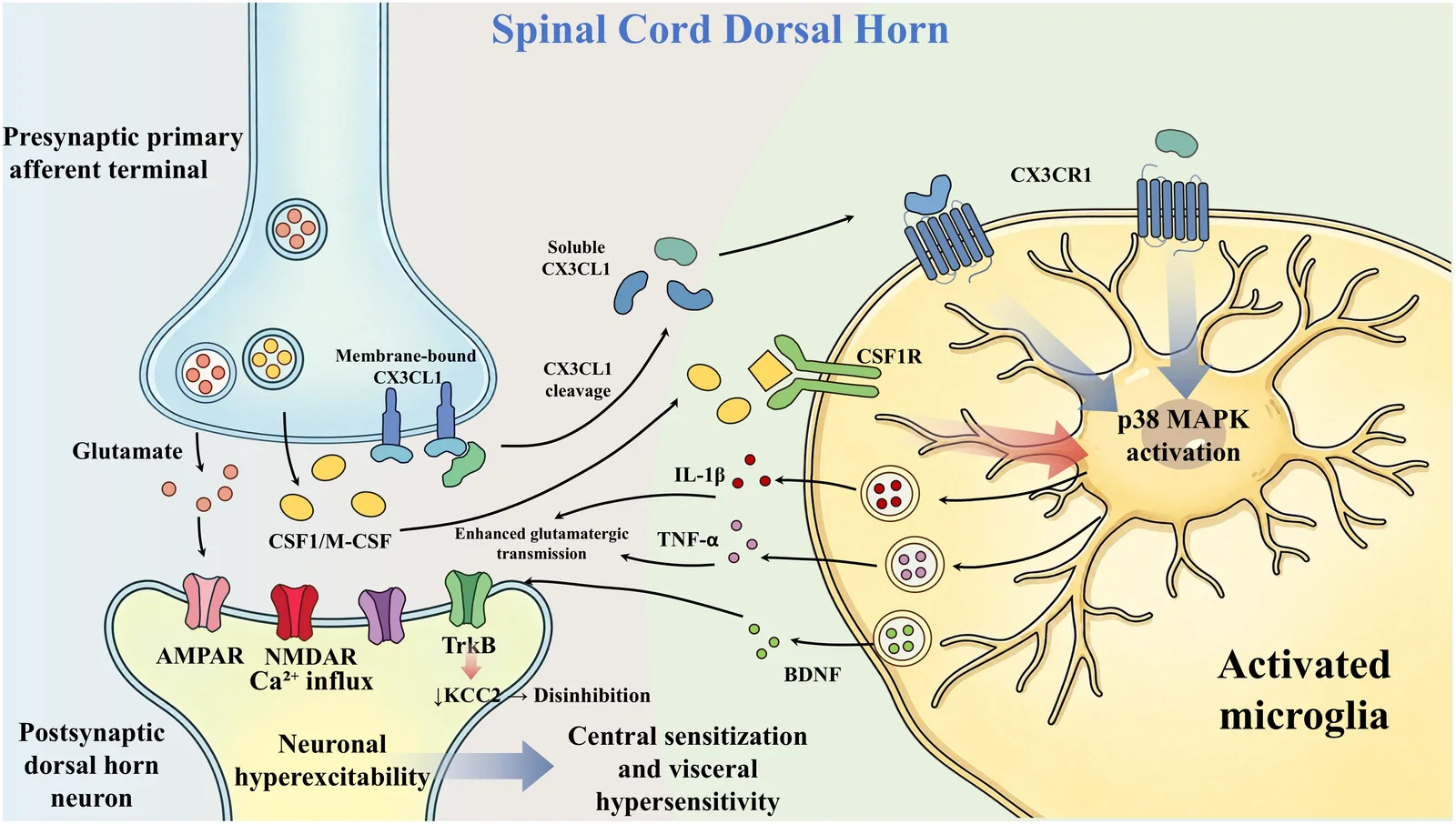
Schematic illustration of neuron- and glia-derived CX3CL1-CX3CR1 and CSF1-CSF1R signaling pathways in spinal microglial activation and visceral pain-related central sensitization. Neuronal membrane-bound CX3CL1 is cleaved into soluble CX3CL1, which activates microglial CX3CR1, while neuron-derived CSF1/M-CSF activates CSF1R; both pathways converge on p38 MAPK activation in microglia. Activated microglia release IL-1beta, TNF-alpha, and BDNF, thereby enhancing glutamatergic transmission and inducing TrkB-dependent KCC2 downregulation in dorsal horn neurons, ultimately promoting neuronal hyperexcitability, central sensitization, and visceral hypersensitivity.

The CSF1-CSF1R signaling pathway is an important mechanism regulating spinal microglial proliferation and activation and plays a critical role in chronic pain-related central neuroimmune responses. Previous studies have shown that in peripheral nerve injury models, CSF1 derived from DRG sensory neurons can act on CSF1R expressed by spinal microglia to promote their proliferation and activation (97). In a DSS-induced colitis-associated VH model, CSF1 derived from spinal astrocytes can also mediate microglial responses and drive VH (24). Activated microglia further release mediators such as IL-1β, TNF-α, IL-6, and BDNF, thereby increasing dorsal horn neuron excitability and promoting central sensitization. These findings suggest that CSF1-CSF1R signaling may contribute to the development and maintenance of visceral pain by promoting spinal glia-glia crosstalk and central sensitization. Together, these findings support a model in which neuron- and glia-derived signals converge on spinal microglia to amplify neuroimmune interactions in the dorsal horn. Persistent visceral nociceptive input may promote the cleavage and release of neuronal CX3CL1, as well as the production of CSF1 from sensory neurons or spinal glial cells. These signals activate microglial CX3CR1 and CSF1R, leading to p38 MAPK activation and the release of proinflammatory and neuromodulatory mediators, including IL-1β, TNF-α, and BDNF. These mediators further enhance glutamatergic transmission, promote AMPAR/NMDAR-mediated Ca^2+^ influx, and impair KCC2-dependent inhibitory control through BDNF-TrkB signaling, thereby increasing dorsal horn neuronal excitability and contributing to central sensitization and visceral hypersensitivity (**Figure 4**).

## 4 Microglia-related signal diffusion and response model

To characterize theoretically how damage-associated signals propagate within the local microenvironment and how they drive microglial activation and downstream changes in neuronal membrane responsiveness, a two-dimensional spatiotemporally coupled model was established using COMSOL Multiphysics 6.4. The Transport of Diluted Species interface was used to resolve the transport of extracellular signaling molecules, and the Domain ODEs and DAEs interface was used to resolve the local state variables, namely the microglial activation variable *m_ga_* and the membrane-response index *V_m_*. Three mediators were represented: CSF1 and ATP as upstream stimulatory signals that diffuse within the tissue microenvironment and progressively establish concentration gradients, and BDNF as the downstream effector released by activated microglia. The model therefore represents the sequential process linking upstream signaling, microglial activation, BDNF release and membrane response. It is a deliberately reduced, idealized two-dimensional representation intended to expose the qualitative spatiotemporal logic of this cascade rather than to provide a quantitatively calibrated representation of dorsal-horn tissue, and the model settings are described below and summarized in **Table 1**.

**Table 1:** Model parameters and numerical settings used in the COMSOL simulations.

Parameter/setting	Description	Value
*D*_CSF1_	CSF1 diffusion coefficient	1.0×10^-10^m^2^s^-1^
*D*_ATP_	ATP diffusion coefficient	5.0×10^-10^m^2^s^-1^
*D*_BDNF_	BDNF diffusion coefficient	1.0×10^-10^m^2^s^-1^
*k*_deg,i_	Distributed degradation coefficient	0 (no distributed sink)
*c*_0,ATP_	ATP input amplitude	50 μm (5.0×10^-2^molm^-3^)
*τ*_ATP,on_	ATP input rise time	60s
*c*_0,CSF1_	CSF1 input amplitude	1.0×10^-15^molm^-3^
*τ*_CSF1,on_	CSF1 input rise time	3,600s
*J*_BDNF,act_	Activation-dependent BDNF release flux	1.0×10^-3^molm^-2^s^-1^
*k*_act,MG_	Microglial activation rate constant	1.0×10^-3^s^-1^
*k*_deact,MG_	Microglial deactivation rate constant	1.0×10^-5^s^-1^
*k*_Vm_	Membrane-response relaxation rate	1.0×10^-4^s^-1^
*MG*_switch_	Microglial activation scenario	0 (non-activated) or 1 (activated)
Geometry	2D planar local microenvironment	Length unit: μm
Mesh	Free triangular, physics-controlled	Local refinement at input/release boundaries
Study and solver	Time-dependent, variable-order BDF	0-300,000s; output every 300s

**Figure 5:**
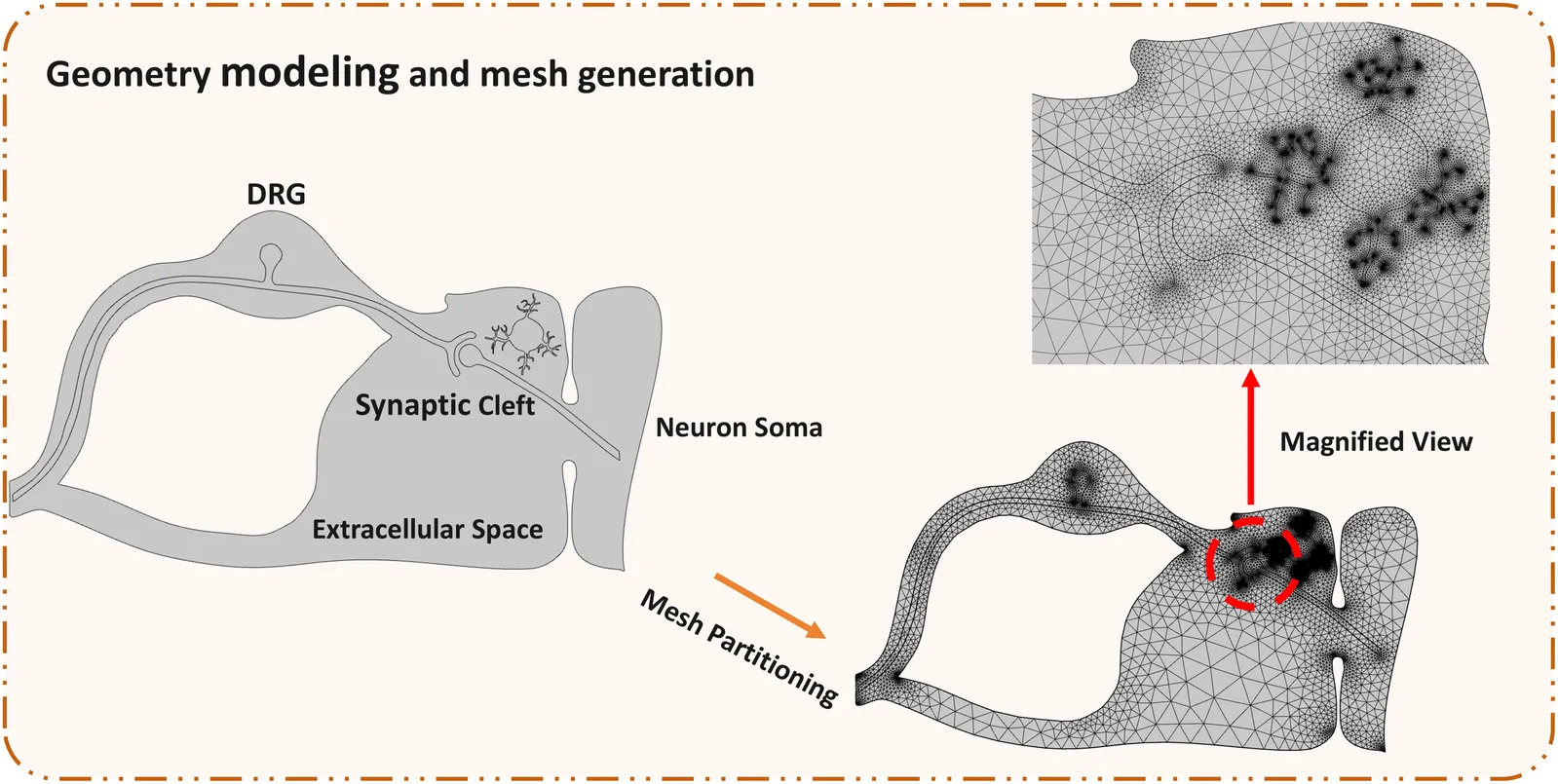
Schematic representation of the established model and the mesh distribution.

Because the objective was to analyze the local diffusion dynamics and spatiotemporal distribution characteristics of damage-associated signals rather than to reproduce complex three-dimensional tissue architecture, the microenvironment was simplified as a two-dimensional planar geometry, with length units defined in μm. **Figure 5** shows the computational domain of the established model together with the mesh distribution. The domain represents a local neuronal microenvironment comprising the dorsal root ganglion region, the neuron soma, the synaptic cleft and the surrounding extracellular space, and boundary conditions were assigned according to biological function, as specified below. The domain was discretized with a free triangular mesh, which accommodates the irregular internal boundaries of the geometry and improves the representation of local concentration gradients. Element sizing was physics-controlled, so that the mesh is automatically refined near the signal input boundary and the BDNF release boundary, where the steepest concentration gradients develop, and remains comparatively uniform in the interior, balancing numerical accuracy against computational cost; the magnified inset in **Figure 5** illustrates the resulting local refinement. The transient problem was solved with the built-in time-dependent solver using variable-order backward-differentiation time stepping with the default relative tolerance, over t=0-3,000,000 s, with solutions stored every 300 s.

In the mathematical description of the model, the spatial transport of CSF1, ATP and BDNF is represented by the general reaction-diffusion form:

(1) $$\frac{\partial c}{\partial t}=D_{i}\nabla^{2}c_{i}-k_{deg,i}c_{i}$$

Where, *c_i_* denotes the concentration of the *i*-th signaling molecule, *D_i_* is the diffusion coefficient, and *k_deg, i_* is the first-order degradation rate constant. Accordingly, the governing equations for CSF1, ATP, and BDNF are expressed as follows:

(2) $$\frac{\partial c_{CSF1}}{\partial t}=D_{CSF1}\nabla^{2}c_{CSF1}-k_{deg,CSF1}c_{CSF1}$$

(3) $$\frac{\partial c_{ATP}}{\partial t}=D_{ATP}\nabla^{2}c_{ATP}-k_{deg,ATP}c_{ATP}$$

(4) $$\frac{\partial c_{BDNF}}{\partial t}=D_{BDNF}\nabla^{2}c_{BDNF}-k_{deg,BDNF}c_{BDNF}$$

(Equations 1–4) state the general transport form. In the simulations reported here the distributed sink term was set to zero, k_deg,i_=0, so that within the domain the three mediators are transported purely diffusively and the characteristic time course of each mediator is determined entirely by its prescribed boundary source term. CSF1 and ATP enter through time-dependent input envelopes that combine a finite rise with a subsequent exponential decay, governed respectively by the rise constants τ_CSF1_, on and τ_ATP_,_on_ and by the decay constants τ_CSF1_,_off_ and τ_ATP_, decay and BDNF is released through a comparable decaying envelope (**Table 1**). Clearance is therefore represented phenomenologically at the source rather than as a distributed sink, which keeps the number of free parameters small and confines the characteristic time scale of each mediator to a single, explicitly stated constant. ATP combines a comparatively high diffusion coefficient with a short, rapidly decaying envelope and therefore acts as an early, transient stimulus, whereas CSF1 diffuses more slowly under a substantially longer envelope and provides a more sustained stimulatory background; BDNF, released by activated microglia, accumulates locally before diffusing into the surrounding region. The model further incorporates a microglial activation variable, *m_ga_*, bounded between 0 and 1, which represents the degree of transition of microglia from a resting state to an activated state. It follows first-order activation and deactivation kinetics driven by the combined CSF1 and ATP stimulus:

(5) $$\frac{dm_{\text{ga}}}{dt}=k_{\text{act},\text{MG}}\;MG_{\text{switch}}\;S_{\text{CSF}1}\left(t\right)\;S_{\text{ATP}}\left(t\right)\;\left(1-m_{\text{ga}}\right)-k_{\text{deact},\text{MG}}\;m_{\text{ga}}$$

Where S_CSF1_(t) and S_ATP_(t) denote the same dimensionless stimulus envelopes that define the CSF1 and ATP inputs, k_act,MG_ and k_deact,MG_ are the activation and deactivation rate constants, and the factor (1-m_ga_) enforces saturation at full activation. The activation variable is thus driven by the prescribed stimulus envelopes rather than by the locally resolved concentration fields. To compare the differences between microglial activation and non-activation conditions, the pathological activation of microglia was switched off as follows:

(6) $$MG_{\text{switch}}=0$$

When pathological microglial activation was enabled:

(7) $$MG_{\text{switch}}=1$$

MG_switch_ is a binary scenario variable that distinguishes the non-activated (MG_switch_ = 0) and activated (MG_switch_ = 1) conditions (Equations 5–7). It gates only the pathological activation-dependent increment: basal BDNF release and the basal membrane response are retained in both scenarios, so that the two conditions differ solely in the activation-dependent contribution.

The membrane response is represented by the index *V*_m_. It should be noted that *V*_m_ does not denote the actual membrane potential in millivolts; it is a dimensionless index of neuronal membrane responsiveness, defined on a normalized scale on which 0 corresponds to no response and 1 corresponds to the maximal response attainable within the model. *V*_m_ relaxes with first-order kinetics towards a target state $V_{\text{m}}^{*}$ that is set by the prevailing microglial activation level. Here *k*_Vm_ is the relaxation rate constant, *V*_m,baseline_ is the response floor in the absence of any microglial contribution, *MG*_basal_ is the basal non-pathological microglial activation level, and *V*_m,gain_ scales the activation-dependent increment. Every quantity on the right-hand side is dimensionless, and the gain is chosen so that $V_{\text{m}}^{*}$ remains within the interval [0, 1]; *V*_m_ is therefore dimensionless by construction. Its dynamic equation describes the temporal evolution of neuronal responsiveness under the influence of microglial activation, without incorporating detailed ion-channel kinetics or action potential generation, and *V*_m_ is evaluated as a domain average over the neuronal domain by means of a domain probe.

Under the boundary conditions, CSF1 and ATP, as upstream input signals, were applied at the designated signal-input boundary; the BDNF release flux was imposed on the microglia-associated boundary and consisted of a basal release term together with a microglial activation-dependent release term. The remaining boundaries were set as no-flux boundaries. This no-flux closure treats the modeled region as a locally isolated microenvironment, which is appropriate over the length and time scales considered here but excludes vascular and cerebrospinal-fluid exchange; the applicability of the model is bounded accordingly. For the initial conditions, all diffusive species were assigned zero background concentration, *m*_ga_ was set close to the quiescent state, and *V_m_* was set to its baseline normalized response level. The parameter values and numerical settings are summarized in **Table 1**.

Within these bounds, the model is intended as a hypothesis-generating framework rather than a substitute for experimental measurement, and this is also where its principal value relative to conventional wet-lab work lies. It resolves continuous concentration fields in space and time, whereas tissue sampling and immunostaining yield sparse and largely terminal snapshots; it permits strictly controlled counterfactual comparisons, such as the *MG*_switch_ = 0 and *MG*_switch_ = 1 scenarios, in which a single causal element is removed while all other conditions are held identical, which is not achievable *in vivo* because pharmacological or genetic manipulation inevitably perturbs multiple pathways simultaneously; it isolates the contribution of individual parameters and thereby indicates which of them dominate the predicted response; and it identifies informative sampling locations and time windows, so that subsequent experiments can be designed more economically and with fewer animals.

### 4.1 Numerical simulation of the microglial activation coupling process

**Figure 6:**
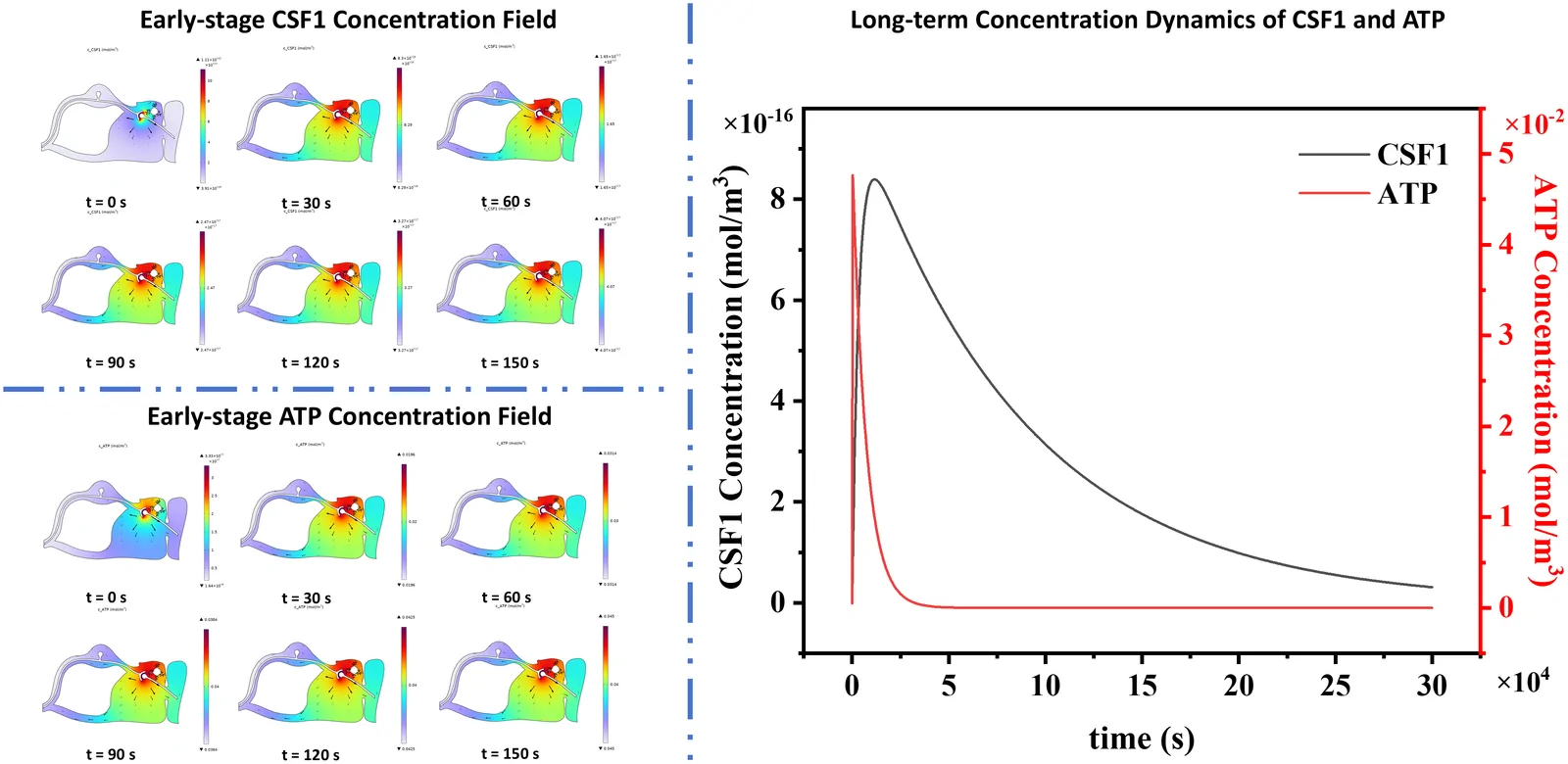
Spatial distribution of CSF1 and ATP early-stage concentration under the MG_switch_=1 condition and long-term Concentration Dynamics of CSF1 and ATP. Left: early CSF1 (upper) and ATP (lower) concentration fields at 0, 30, 60, 90, 120, and 150 s, showing diffusion from the local release site. Right: long-term concentration curves from 0 to 3.0×10^5^ s; CSF1 is shown in black using the left y-axis, and ATP is shown in red using the right y-axis.

Based on the established COMSOL Multiphysics model, this section further investigates the spatiotemporal dynamics of CSF1, ATP, and BDNF under the *MG*_switch_=1 condition. Furthermore, by comparing the normalized membrane response *V*_m_ between the *MG*_switch_=0 and *MG*_switch_=1 conditions, the impact of microglial activation on neuronal responsiveness is examined. **Figure 6** shows the early-stage spatial concentration distributions of CSF1 and ATP under the MG_switch_=1 condition, as well as their long-term concentration dynamics. Under the *MG*_switch_=1 condition, CSF1 and ATP are released as upstream signaling molecules and diffuse within the local microenvironment. The early concentration maps show that both mediators spread from the local release site into the surrounding tissue and form higher-concentration regions near the source. The long-term concentration curves showed distinct temporal profiles for CSF1 and ATP. CSF1 increased from near baseline to a peak of approximately 8.4×10^−16^mol/m^3^ at approximately 1.2×10^4^s and then gradually declined to approximately 3.0×10^-17^mol/m^3^ at 3.0×10^5^s. In contrast, ATP rapidly reached a maximum concentration of approximately 4.7×10^-2^mol/m^3^ during the early phase of the simulation, declined to less than 10% of its peak within approximately 1.2×10^4^s, and approached baseline by approximately 3.0×10^4^s. These results indicate that ATP primarily provides an early and transient initiating signal, whereas CSF1 provides a more sustained microglial activation background.

**Figure 7:**
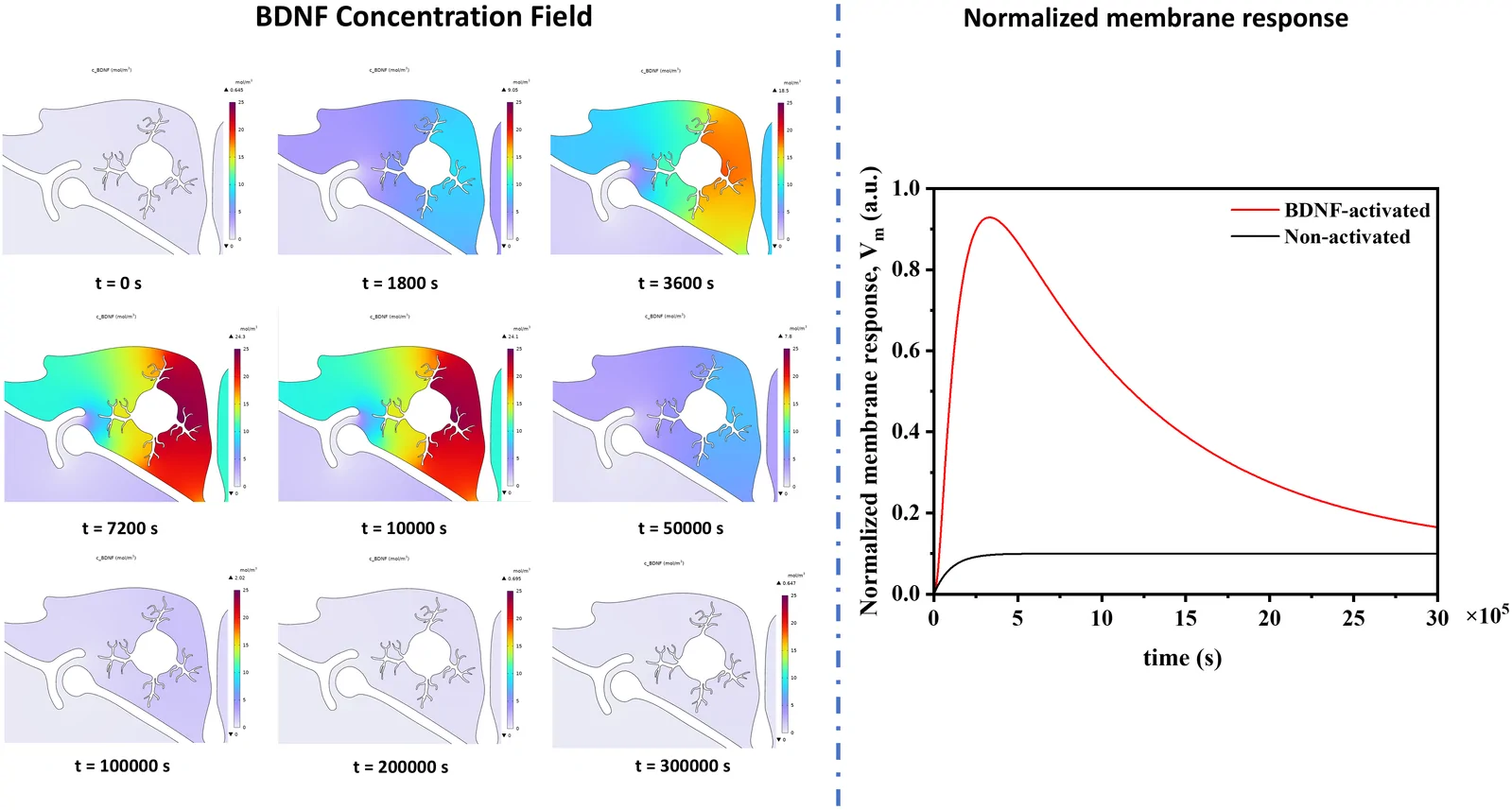
Spatial distribution of BDNF concentration under the MG_switch_=1 condition and time course of the dimensionless membrane-response index V_m_ under the MG_switch_=0 and MG_switch_=1conditions. The left panels show the spatial distribution of BDNF concentration under the *MG*_switch_=1 condition at 0, 1,800, 3,600, 7,200, 10,000, 50,000, 100,000, 200,000, and 300,000s, demonstrating local accumulation, diffusion, and subsequent attenuation of BDNF. The right panel shows the time course of the dimensionless membrane-response index *V*_m_ from 0 to 3.0×10^6^s. The activated condition (red curve) reaches a peak *V*_m_ of approximately 0.920 at approximately 3.2×10^5^s, whereas the non-activated condition (black curve) remains near 0.091, corresponding to an approximately 10.10-fold difference at the peak-response time point.

Under the combined stimulation of CSF1 and ATP, microglial activation increases and is accompanied by BDNF release. **Figure 7** shows the spatial distribution of the BDNF concentration field under the *MG*_switch_=1 condition, together with the temporal changes in the membrane-response index *V*_m_ under the *MG*_switch_=0 and *MG*_switch_=1 conditions. The BDNF concentration maps show local accumulation near the microglia-associated release site, followed by diffusion into the surrounding tissue and gradual attenuation at later time points. Further comparison of the temporal changes in *V*_m_ shows that the membrane-response index remained at a low basal level of approximately 0.091 under the *MG*_switch_=0 condition. In contrast, under the *MG*_switch_=1 condition, *V*_m_ increased markedly and reached a peak value of approximately 0.920 at 3.2×10^5^s, representing an approximately 10.10-fold increase relative to the non-activated condition. Although the activated response gradually declined after reaching its maximum, it remained approximately 0.16 at 3.0×10^6^s, which was still approximately 1.76-fold higher than the non-activated response.

Because the model is deterministic, repeated evaluation of the same parameter set returns identical trajectories, and conventional statistical descriptors such as standard deviation, standard error or confidence intervals are not applicable to this ratio: the formulation contains no stochastic term and no biological replicate structure from which such variability could arise. The uncertainty attached to the ratio is parametric rather than statistical. Its magnitude is governed principally by the three parameters that set the denominator, namely the response floor *V*_m,baseline_ together with the gain *V*_m,gain_ and the basal activation level *MG*_basal_, so that the ratio increases as the basal response decreases. It is by contrast insensitive to the diffusion coefficients, because the activation variable *m*_ga_ is driven by the prescribed CSF1 and ATP stimulus envelopes rather than by the locally resolved concentration fields, and *V*_m_ depends on the mediators only through *m*_ga_. The value should accordingly be read as a trend-level model output indicating the direction and approximate scale of the predicted effect under the stated assumptions, rather than as a measured biological effect size. For the same reason, because the *V*_m_ equation is driven by *m*_ga_ rather than by an explicit *c*_BDNF_- or TrkB-dependent term, BDNF accumulation and the rise in *V*_m_ are parallel consequences of microglial activation within the model, and the figure supports an association between microglial activation and enhanced membrane responsiveness rather than a directly resolved BDNF-to-*V*_m_ causal chain. Within these limits, the results indicate that microglial activation can markedly enhance neuronal membrane responses, suggesting increased neuronal excitability and thereby potentially contributing to the exacerbation of neuropathic pain.

## 5 Therapeutic opportunities and translational significance

Elucidating the central role of microglia in visceral pain signal transmission and spinal sensitization not only advances our understanding of the pathophysiological mechanisms underlying chronic visceral pain, but also provides new perspectives for the development of targeted interventions that integrate both peripheral and central mechanisms. Based on current evidence, potential therapeutic opportunities mainly include the following aspects. Beyond these mechanism-based therapeutic targets, the clinical translation of microglia-related mechanisms in chronic visceral pain will likely depend on the development of biomarker-guided frameworks for patient stratification and individualized treatment. In this context, circulating, imaging, molecular, and other emerging biomarkers may help define distinct neuroimmune and central sensitization phenotypes, thereby supporting precision pain medicine and improving the applicability of targeted interventions (98).

### 5.1 Targeting microglial activation and upstream signaling

#### 5.1.1 Purinergic receptor antagonists

Microglial purinergic receptors are important upstream regulatory nodes in neuroinflammation associated with chronic pain. P2X4 and P2X7 receptors primarily sense extracellular ATP, whereas P2Y12 receptors mainly respond to ADP; all three receptor subtypes can contribute to microglial activation and enhanced excitatory synaptic transmission in the spinal dorsal horn. In models of neuropathic pain and inflammation-related pain, antagonism of these receptors alleviates neuroinflammation and pain hypersensitivity, suggesting their potential therapeutic value (99, 100). Particularly in the field of visceral pain, studies have shown that blockade of spinal P2X4 receptors significantly ameliorates VH in rats subjected to neonatal maternal separation (101), indicating that targeting microglial purinergic signaling may provide a novel strategy for the treatment of chronic visceral pain.

#### 5.1.2 Blockade of CX3CL1/CX3CR1 signaling

The CX3CL1/CX3CR1 signaling pathway is an important regulatory axis in neuron-microglia communication. In models of neuropathic pain, blockade of this pathway or inhibition of CX3CL1 cleavage and release attenuates microglial responses and pain-related behaviors, suggesting its therapeutic potential (102). Studies have also shown that, in models of VH, spinal cathepsin S promotes VH through the FKN/CX3CR1/p38 MAPK pathway, indicating that blockade of this signaling axis may represent a potential strategy for the treatment of chronic visceral pain (26). In addition, CX3CR1 is involved in intestinal macrophage-mediated mucosal immune regulation and inflammatory processes in IBD (103), suggesting that interventions targeting this pathway may affect both peripheral intestinal inflammation and central neuroimmune processes; however, its tissue-specific effects require further evaluation.

#### 5.1.3 Inhibition of CSF1/CSF1R signaling

CSF1/CSF1R signaling plays an important role in post-injury microglial proliferation and the maintenance of a sensitized state. Sensory neuron-derived CSF1 can activate CSF1R on spinal microglia, promoting their expansion and inflammatory responses, thereby driving dorsal horn sensitization and pain maintenance (97). Intervention in this pathway may attenuate microglia-mediated neuroinflammation and pain hypersensitivity. Notably, CSF1R inhibitors have entered the drug development landscape in the field of cancer immunotherapy, suggesting that this target has translational potential (104). However, their application in chronic visceral pain requires further evaluation, particularly regarding safety and tissue-specific effects.

### 5.2 Modulation of synaptic plasticity and excitation/inhibition balance

Central sensitization in the spinal dorsal horn is characterized not only by enhanced excitatory synaptic plasticity mediated by glutamate-NMDAR signaling, but also by weakened GABAergic/glycinergic inhibition resulting from KCC2 downregulation. These two processes mutually reinforce each other, jointly leading to an excitation/inhibition imbalance in dorsal horn neural circuits and sustaining VH. Therefore, directly inhibiting aberrant NMDAR activation or correcting BDNF-TrkB-KCC2-mediated disinhibition may have potential therapeutic value (74, 83, 105, 106).

#### 5.2.1 NMDAR antagonists

NMDARs constitute an important molecular basis for activity-dependent synaptic plasticity and central sensitization in the spinal cord. Persistent or repeated nociceptive input can relieve the Mg^2+^ block of NMDARs, promoting Ca^2+^ influx and activating downstream signaling pathways such as CaMKII, PKC, and ERK. These events enhance the excitability of dorsal horn neurons and induce LTP-like changes. Experimental studies have shown that NMDAR antagonists, such as ketamine, can attenuate spinal sensitization and pain hypersensitivity (107–109). Clinically, ketamine has shown short-term analgesic potential in some patients with refractory pain; however, its use is limited by psychotomimetic symptoms, sedation, cardiovascular adverse effects, and limited durability of analgesic benefit. Therefore, for chronic visceral pain, NMDAR antagonism is currently more appropriately positioned as an adjunctive intervention for severe acute exacerbations, perioperative pain management, or cases refractory to conventional therapies, rather than as a long-term maintenance treatment (110–112).

#### 5.2.2 Targeting the BDNF-TrkB-KCC2 axis to correct the inhibition-excitation imbalance

In neuropathic pain, BDNF released by activated microglia acts on TrkB receptors in dorsal horn neurons, downregulating KCC2 expression or function, disrupting Cl^-^ homeostasis, weakening GABAergic/glycinergic inhibition, and leading to spinal disinhibition. This process can also lower the neuronal excitation threshold and amplify NMDAR-dependent synaptic plasticity, thereby sustaining central sensitization (37, 81, 84). This mechanism has also attracted attention in some models of VH, suggesting that blocking TrkB signaling, restoring KCC2 function, or enhancing GABAergic/glycinergic transmission may alleviate pain by correcting the inhibition-excitation imbalance (25, 75). However, their clinical translation will require greater spatiotemporal selectivity and cell-type specificity. Most current evidence is still derived from animal models, and human data remain limited. Recent single-cell studies also suggest that microglial phenotypes are highly heterogeneous, indicating that further translational validation is needed.

## 6 Conclusion

Increasing evidence indicates that spinal microglial activation and microglia-mediated neuroinflammation are not merely concomitant phenomena in chronic visceral pain, but are more likely to represent key mechanisms driving the development and maintenance of VH. Under persistent visceral nociceptive input, microglia in the spinal dorsal horn shift from a homeostatic surveillant state to an activated phenotype and release a range of proinflammatory cytokines, chemokines, and neurotrophic factors, thereby reshaping the local neuroimmune microenvironment. These changes can enhance excitatory synaptic transmission, weaken inhibitory control, promote aberrant synaptic plasticity and central sensitization, and ultimately lead to amplification and chronification of visceral pain signaling. Among these mechanisms, purinergic signaling, the CX3CL1-CX3CR1 pathway, the CSF1/CSF1R pathway, and effector molecules such as TNF-α, IL-1β, IL-6, and BDNF all participate in the functional remodeling of spinal nociceptive circuits. Notably, substantial evidence from neuropathic pain studies has shown that microglia-derived BDNF can induce KCC2 downregulation through TrkB and enhance NMDAR-related signaling. Similar mechanisms have also attracted attention in some models of VH, suggesting that they may promote the establishment and maintenance of central sensitization by inducing disinhibition and synaptic potentiation. Overall, spinal microglia occupy a pivotal position linking peripheral inflammation, neuroimmune activation, and central amplification of pain.

From a translational medicine perspective, interventions targeting microglial activation and its key signaling pathways provide new therapeutic avenues for IBS, IBD, and other chronic visceral pain-related disorders. Targeting P2X/P2Y receptors, CX3CL1/CX3CR1, CSF1/CSF1R, and BDNF-TrkB-KCC2/NMDAR-related pathways may help suppress spinal neuroinflammation, reverse central sensitization, and improve symptoms associated with VH. Therefore, systematically elucidating the molecular and cellular mechanisms underlying spinal microglial activation will not only deepen our understanding of the neuroimmune pathological basis of chronic visceral pain, but also provide an important theoretical foundation for the development of precision therapeutic strategies. However, most current evidence is still derived primarily from animal experiments and preclinical studies, and the clinical efficacy, target selectivity, and long-term safety of these approaches require further validation.

Future studies should further investigate the heterogeneity of spinal microglia in chronic visceral pain, with particular emphasis on their spatiotemporal characteristics and functional states during the initiation, maintenance, and resolution of VH. Single-cell transcriptomics, spatial transcriptomics, and proteomics may help identify microglial subpopulations associated with inflammatory responses, synaptic remodeling, and pain regulation. Meanwhile, more physiologically relevant three-dimensional (3D) coupled models, including spinal cord organoids, organ-on-a-chip platforms, and gut-spinal cord or gut-brain-spinal cord models, are needed to better reproduce the complex interactions among neurons, glial cells, immune cells, and gut-derived factors and to provide platforms for drug screening and target validation. From a translational perspective, the blood-spinal cord barrier remains a major obstacle to drug delivery; therefore, strategies such as nanoparticle-based carriers, ligand-mediated delivery, extracellular vesicles, and local or stimulus-responsive delivery systems should be explored to improve spinal targeting and minimize off-target effects. In addition, sex, hormonal status, disease stage, gut dysbiosis, psychological stress, gastrointestinal inflammation, and abnormal mucosal immune responses may all influence microglial activation and neuroimmune signaling through the gut-brain axis. Integrating peripheral pathological alterations with spinal neuroimmune mechanisms, while taking into account microglial heterogeneity, sex differences, and challenges in drug delivery, will help advance our understanding of VH and facilitate the clinical translation of microglia-targeted therapies.
